# Locality-sensitive hashing enables efficient and scalable signal classification in high-throughput mass spectrometry raw data

**DOI:** 10.1186/s12859-022-04833-5

**Published:** 2022-07-20

**Authors:** Konstantin Bob, David Teschner, Thomas Kemmer, David Gomez-Zepeda, Stefan Tenzer, Bertil Schmidt, Andreas Hildebrandt

**Affiliations:** 1 Institute of Computer Science, Johannes Gutenberg University Mainz, D-55128 Mainz, Germany; 2 Institute for Immunology, University Medical Center of the Johannes Gutenberg University Mainz, D-55128 Mainz, Germany; 3 Immunoproteomics Unit, Helmholtz-Institute for Translational Oncology (HI-TRON) Mainz, D-55131 Mainz, Germany

**Keywords:** Mass spectrometry, Locality-sensitive hashing, Signal processing

## Abstract

**Background:**

Mass spectrometry is an important experimental technique in the field of proteomics. However, analysis of certain mass spectrometry data faces a combination of two challenges: first, even a single experiment produces a large amount of multi-dimensional raw data and, second, signals of interest are not single peaks but patterns of peaks that span along the different dimensions. The rapidly growing amount of mass spectrometry data increases the demand for scalable solutions. Furthermore, existing approaches for signal detection usually rely on strong assumptions concerning the signals properties.

**Results:**

In this study, it is shown that locality-sensitive hashing enables signal classification in mass spectrometry raw data at scale. Through appropriate choice of algorithm parameters it is possible to balance false-positive and false-negative rates. On synthetic data, a superior performance compared to an intensity thresholding approach was achieved. Real data could be strongly reduced without losing relevant information. Our implementation scaled out up to 32 threads and supports acceleration by GPUs.

**Conclusions:**

Locality-sensitive hashing is a desirable approach for signal classification in mass spectrometry raw data.

**Availability:**

Generated data and code are available at https://github.com/hildebrandtlab/mzBucket. Raw data is available at https://zenodo.org/record/5036526.

**Supplementary Information:**

The online version contains supplementary material available at 10.1186/s12859-022-04833-5.

## Background

### Mass spectrometry in proteomics

Valuable information for medicine and the design of new drugs for several severe diseases [1] are expected to be gained by new discoveries in proteomics [2–4], the field that studies proteins experimentally on a large scale. An experimental technique commonly used in proteomics is mass spectrometry (MS) [5], which allows to separate ionized molecules by their mass-to-charge ratio (m/z) and which can be combined with measurement of other physical and chemical properties.

The overall goal in an untargeted MS-based proteomics experiment is to identify and quantify as many proteins as possible in a given sample with a high quantitative performance in terms of precision and reproducibility. In particular, in bottom-up proteomics proteins are digested into peptides first and then those peptides are measured.

**Fig. 1:**
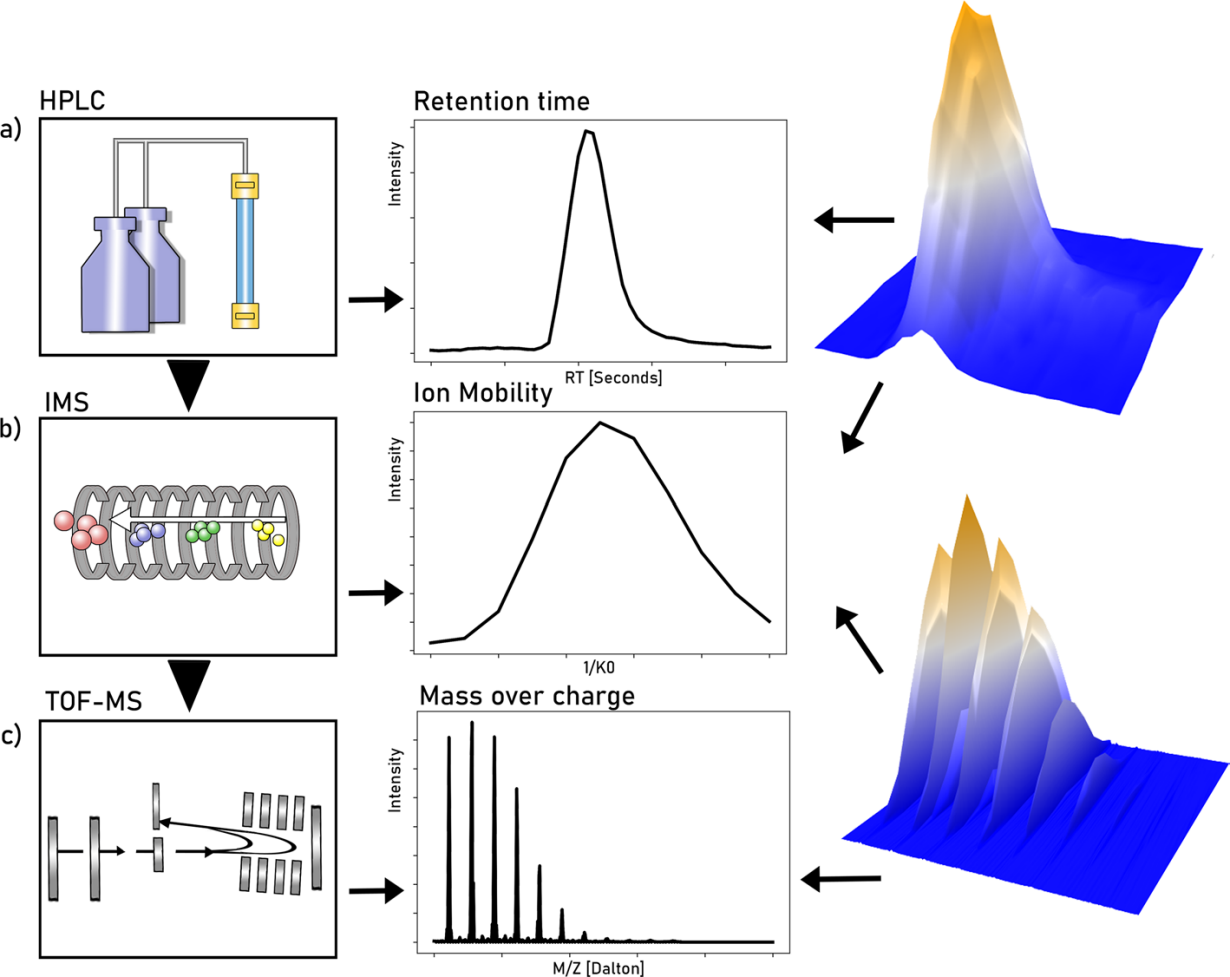
Experimental workflow in an LC-IMS-MS setup and resulting signal of interest. **a** Chemical separation by high-performance liquid chromatography (HPLC) and resulting intensity distribution along retention time. **b** Subsequent ion-mobility separation (IMS) and resulting intensity distribution along the mobility dimension. **c** Time-of-flight mass spectrometry (TOF-MS) and resulting isotopic pattern, i.e., evenly spaced peaks with a certain distribution of the peaks envelope. The column on the right shows the signal as a function of two variables, drift time and retention time on the top and mass-over-charge ratio and drift time on the bottom. Note the repeated occurrence of these isotopic patterns in the 3D plot on the bottom right

If only the mass-to-charge ratio of a large, complex sample of peptides was measured, the resulting signal would be highly convoluted as many peptides have the same or a very similar m/z. In order to minimize overlapping signals and to get further information on the molecules measured, mass spectrometers are coupled with a previous separation device based on orthogonal (ideally) physical and chemical properties. Typically, the peptides are first separated using liquid chromatography (LC), where molecules are separated and gradually eluted at a certain retention time range, e.g., based on their polarity in the commonly used reversed phase (RP) chromatography. More recently, ion mobility separation (IMS) has become widely accessible in commercial mass spectrometers as an extra dimension of separation, where ionized molecules are continually separated based on their shape and size before being analyzed in the MS. For instance, in LC-IMS-MS [6–8] the retention time and mobility dimensions are recorded in addition to m/z. Figure 1 shows the experimental workflow in the left column. Finally, in many experimental setups two types of mass spectra are recorded: so-called MS1 spectra of all the ions, typically for localization of signals of interest denominated precursors and MS2 spectra, where selected (or unselected) precursors are fragmented and the obtained ion patterns (fragmentation spectra) are typically used for identification.

The first step in the analysis of MS1 spectra is to identify regions of interest. These signals form characteristic patterns in the raw data. More precisely, molecules produce so-called isotopic patterns [9] that are caused by the occurrence of different isotopes in the chemical elements. Thus, one expects a pattern of evenly spaced peaks, where the distance between consecutive peaks varies inversely according to the charge state of the molecule (i.e., a spacing of $\approx 1 \mathrm {m/z}$ for $z=1$, $\approx 0.5 \mathrm {m/z}$ for $z=2$, etc.).

The distribution of peak intensity within an isotopic pattern depends on the chemical elements present in a molecule and their respective distribution of isotopes. Although it is possible to deal with the resulting combinatorial complexity [10], often simpler approaches that assume a typical chemical composition are used to model the intensity distribution of the underlying isotope pattern. A common example is the so-called *averagine* model [11].

### Problem statement and challenges

Due to the progressive elution of molecules from the LC and ions from the IMS before reaching the MS analyzer, the signals of interest are expected to occur repeatedly over time with the same pattern along the mass axis. Figure 1 shows an example of a resulting signal in the center and right column.

The problem to be solved can now be formulated as follows: given MS1 spectra of a high-throughput setup with additional dimensions of separation, classify whether a given set of peaks is a signal of interest or not. This formulation is deliberately more general than a concrete task like deisotoping or charge state detection and should be considered as a preceding step to the other tasks.

**Fig. 2:**
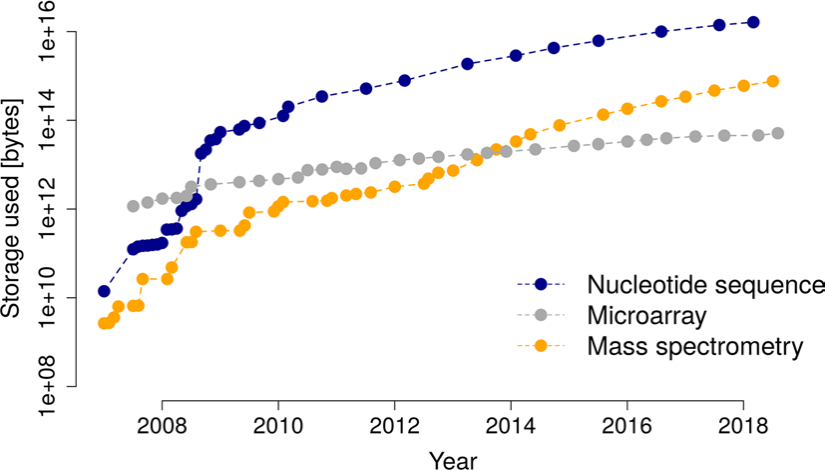
Size of recorded data at the European Bioinformatics Institute (EMBL-EBI) over time for different platforms in life sciences. Note the logarithmic scale on the y-axis. Mass spectrometry data has shown an exponential growth. Plot recreated after [36]

On top of the signal processing challenge, another technical problems arises: by introducing additional dimensions of separation (such as LC and IMS), the sizes of single data sets increase. Combined with a growth in the number of data sets measured, the storage used for mass spectrometry data has thus grown tremendously over the past years (cf. Fig. 2) and is expected to grow further. Consequently, dealing with mass spectrometry raw data could strongly benefit from the usage of Big Data technologies, see, e.g., [12]. In a Big Data approach, data is stored and processed in a distributed manner, which in turn restricts the algorithms applicable.

### Locality-sensitive hashing

An often used Big Data method for the comparison of high-dimensional data is locality-sensitive hashing (LSH) [13, 14]. In particular, it is a generic algorithm for finding similar pairs of data points (by some measure) in linear runtime. However, this reduction of runtime comes at the cost of the algorithm being probabilistic in nature.

Owing to its wide applicability, the technique is widely used in different fields, including image retrieval [15], pattern recognition [16], and genome analysis [17, 18].

### Related work

In the particular context of mass spectrometry, LSH has been used for looking up peptide sequences in databases [19, 20], to cluster different spectra for MS1 spectra on LC-MS data [21], and for fast database lookup on MS2 spectra [22].

Previous approaches to signal detection in mass spectrometry raw data either rely on assumptions concerning the isotopic distribution [23] or are based on deep learning [24] and thus lack interpretability.

Concerning the processing of larger data sets, established tools like MaxQuant [25] partially bypass large data sizes by using only parts of the data, i.e., by only looking at every 4th spectrum by default [26].

To the best of our knowledge, signal classification by means of LSH for mass spectrometry raw data has not been treated publicly.

## Results

Here, we present two approaches to the problem stated above which leverage LSH, both for practical applications and to illustrate the versatility of our work. The first one uses self-similarity of the windows only and is therefore very general. The second one uses reference patterns and therefore relies more heavily on model assumptions. However, in turn one is able to obtain mass and charge state estimates.

### Denoising using self-similarity

Our first approach exploits the fact that all signals originated from true molecular ions within detectable limits will form a signal of interest that repeats across certain consecutive time points [26], while noise is assumed to be much more random. Thus, classification of the signal is achieved by deciding whether there are similar signals present. Finding similar objects is achieved by locality-sensitive hashing, as it allows to leverage parallel computation.

**Fig. 3:**
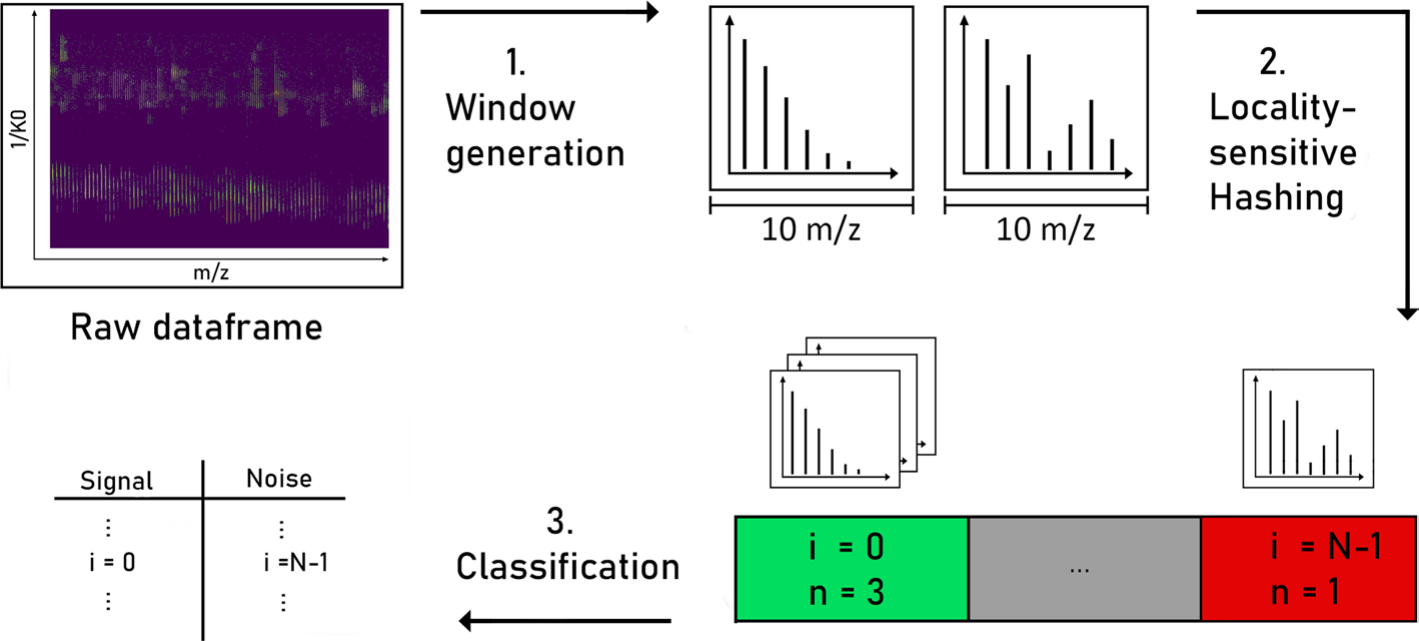
Schematic overview of the approach: short intervals (windows) of the several mass axes are considered as smallest building blocks of the data set and generated from a raw dataframe. Then for each window the (several) hash functions *h* map into the *N* hash buckets (colored boxes at the bottom right). These are indexed from $i=0$ to $i=N-1$ and the number of windows *n* within a bucket is counted. Finally, if more than one window is mapped in a given hash bucket, all windows inside the box are considered “true” signal

The overall scheme of the approach, see Fig. 3 for illustration, is the following: a mass spectrometry raw data set is considered to be a set of mass axes for each possible replicated measurement, that is, each retention time for LC-MS data or each retention time and mobility measurement for LC-IMS-MS data, respectively. These mass axes are then cut into small intervals, called windows. For each window several hash values are computed. If two windows have at least one equal hash value resulting from the same hash function they are said to collide. The classification into “true” signal and noise used the following criterion: if a peak lies within a window that collided with any other window it is considered “true” signal, otherwise noise. To facilitate the lookup of collisions, a second map structure is used that maps hash values to their respective number of occurrences.

A detailed explanation of window generation and hashing functions is given in the Section ’Methods’. As the hash function of each window can be computed and checked independently of the other windows, the algorithm is embarrassingly parallel in nature. By using an augmented LSH with *n* AND connectives and *m* OR connectives, the algorithm allows for tuning of false-positive and false-negative rates.

In particular, our approach assumes no model or distributions for the signal shapes, only similarity. Thus, we are able to distinguish different isotopic patterns as true signal, regardless of the composition, size, and charge of the ionized molecule.

#### Signal classification capability on synthetic data

**Fig. 4:**
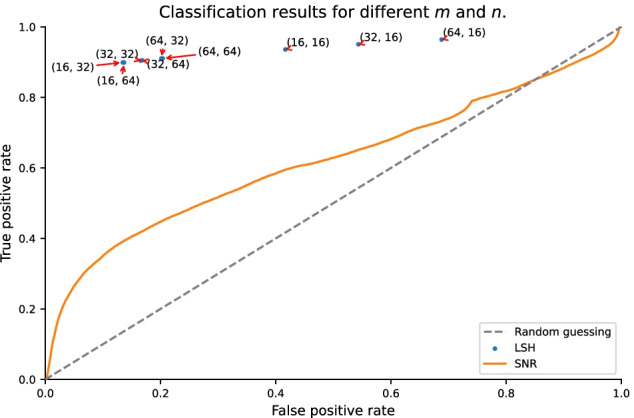
Receiver operating characteristic curve. This plot shows the relative specificity/sensitivity results of two tested signal detection methods LSH and noise threshold (SNR). The dashed line is for reference and shows the results of random guessing

Figure 4 shows the receiver operating characteristic (ROC) of a classification task on synthetic data. By varying the amplification parameters *m* and *n* of the LSH, different types of discrimination can be achieved, depending on the goal of the user^1^.

**Fig. 5:**
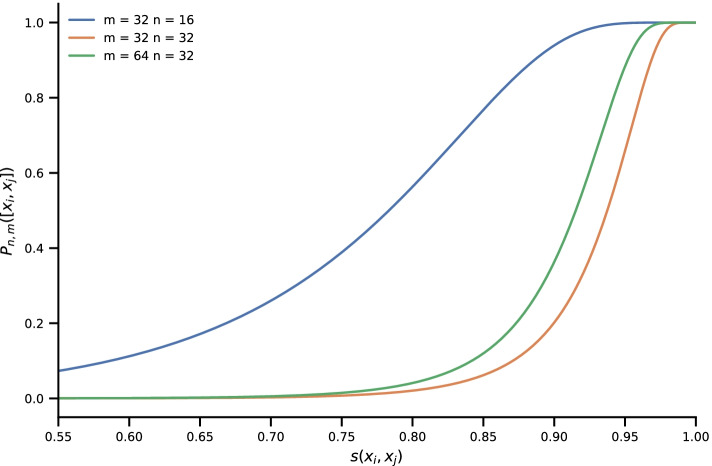
Controlling typical similarity of pairs: probability $P_{m,n}([x_{i},x_{j}])$ to retrieve a pair for several combined hashes as a function of similarity $s(x_{i},x_{j})$. By appropriate choice of *m* and *n*, found pairs have a high probability of having at least a certain similarity. Note that the x-axis starts at $s(x_{i},x_{j})=0.55$

The achieved performance is in accordance with expected behavior when comparing the implied similarity thresholds in Fig. 5. When setting a low threshold, e.g., $(m,n) = (32,16)$ (corresponding to the blue line in Fig. 5), a large fraction of windows are considered signal, resulting in very high true-positive and false-positive rates.

A stricter discrimination with $(m,n) = (64,32)$ improves the performance significantly. This in turn indicates that the typical similarity measure of the data is in the area where the green line in Fig. 5 has a high slope.

Using a very high threshold, e.g., $(m,n) = (32,32)$ (corresponding to the orange line in Fig. 5), some windows are lost, resulting in a lower true-positive rate. As the false-positive rate shrinks as well, this setting may be employed as a strong filter to reduce large data sets.

For further information on the behavior, a heatmap plot varying both *m* and *n* is shown in the Additional file 1.

The performance of our approach was compared to the common approach of filtering signal by an intensity threshold. While the overall behavior of its ROC curve is similar to the one of our approach, the performance is considerably better.

Several synthetic data sets with different noise characteristics were created and all main findings persisted, see Additional file 2 for details.

#### Filtering capability on real data

**Fig. 6:**
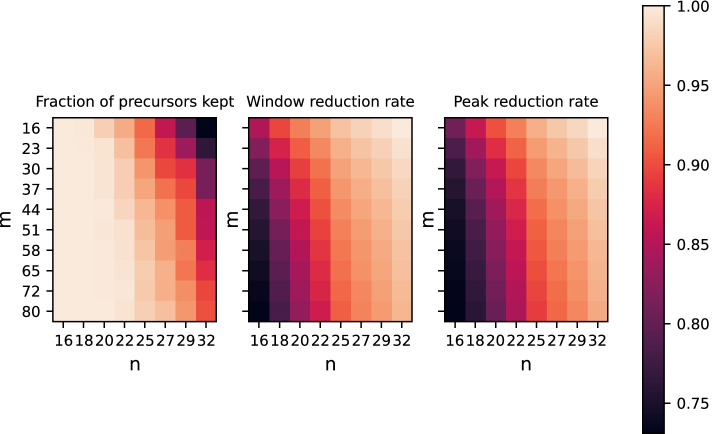
Evalutation on real data: fraction of precursors kept (left panel), window reduction rate (center panel), and peak reduction rate (right panel) as functions of *m* and *n*. For certain choices of *m* and *n* only few windows containing selected precursors are lost, while the data reduction rates in terms of windows and peaks are very high, emphasizing the intended usage of our approach as a prefilter. However, *m* and *n* can also be varied to balance the trade off between recovery rate and reduction

Next, we conducted a study to determine the capability of our approach on real data. An option would have been to use a file with peptides identified by any protein identification software, such as MaxQuant. Nevertheless, this would introduce a bias towards high-quality ions and only those present in the protein database would be considered. Therefore, we decided to employ the precursors fragmented during the DDA-PASEF analysis, which are selected based on the ion charge, intensity, and ion mobility profile. We computed the rate of windows with selected precursor ions kept by our filtering and the reduction in data set size, once in terms of windows and once in terms of peaks, after the filtering, as shown in Fig. 6. As can be observed from the same figure, the reduction rate has to be balanced against the loss of relevant signal by choosing suitable values of *m* and *n*. That way it is possible to reduce the amount of data points in a frame by at least half, while retaining almost all vendor-selected peaks. We found both the reduction rate and the rate of selected precursor ions kept to be between approximately 70% and almost 100%, where the two are inversely related, i.e., higher reduction rate implies lower keeping rate and vice versa. The reduction rate in terms of windows shows the same behavior as the one in terms of peaks.

For details on the evaluation see the Section ’Methods’.

### Using averagine modeled reference patterns for denoising and deisotoping

Translating slices of mass spectra into a set of representative keys is not limited to self-similarity detection. Other approaches used LSH for fast candidate identification of fragment spectra, see for example [21, 22]. There, first a look-up database is built by mapping all reference spectra into LSH-key representations. By using LSH, unidentified candidate spectra only need to be matched with a subset of candidates in the database, greatly reducing the total number of comparisons. For a more detailed explanation of algorithmic solutions to spectral identification in general, the interested reader is referred to [27].

In our second approach, we adapted the general idea of a pre-computed reference, but used typical isotope patterns to find peptide ions of a specific charge state in precursor spectra in contrast to fragment spectra. These synthetic isotope patterns are hashed to form a reference database, which then can be used to detect isotope patterns by collision of hashes in sets of spectrum windows. By design, the approach is more restrictive than our first presented strategy of self-collision. This is due to the fact that it requires a window to be similar to one of the modeled peak distributions.

We used a subset of the real data set mentioned before. The vendor software selected $N=6902$ peptides for fragmentation during DDA-PASEF in that subset. We were able to compare those with isotopic patterns found by our approach. When *m* and *n* are set accordingly, $k_1=6200$ of those $N=6902$ peptides were considered matched by our initial metric ($\approx \,$91% of *N*). $k_2=5125$ of the matches $k_1$ had the correct charge state ($\approx \,$74% of *N*). $k_3=3419$ of the matches $k_2$ perfectly matched the monoisotopic $\mathrm {m/z}$ and charge state ($\approx \,$50% of *N*).

For details on the evaluation see the Section ’Methods’.

### Scalability and acceleration

**Fig. 7:**
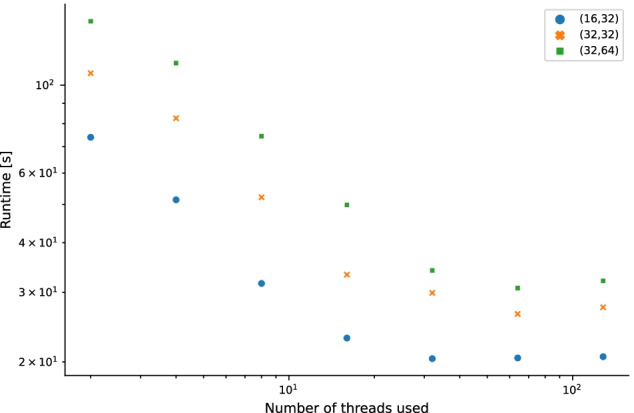
Scalability for different *m* and *n*: wall-clock time of the implementation in seconds as a function of the number of threads used. More precisely, the cumulative runtime for processing of 100 randomly selected precursor frames is shown. Note the logarithmic scales on both axes. First the wall-clock time is a decreasing function of the number of threads used and then saturates. This shows that the implementation scales out well up to a certain point, at which the parallelization overhead cancels the gain in speed. See the text for the setup used

Figure 7 shows the scalability of the approach for different choices of *m* and *n* on parts of a measured data set. In this double-logarithmic plot, the wall-clock time of the implementation is at first a decreasing function of the number of threads used and then saturates. This shows that the implementation scales out well up to a certain point, at which the parallelization overhead cancels the gain in speed. For details regarding the setup, see the Section ’Methods’.

Furthermore, our implementation supports acceleration by graphics processing units (GPUs) through the TensorFlow 2 software library [28].

### Software design and availability

We built on top of the low-level C++ API of OpenTIMS [29] for data access. Furthermore, we used pybind11 [30] to interface C++ with the Python programming language and provide both the C++ and the Python code as a Python package. This facilitates the integration of our tool into the well-established, Python-centric data science stack.

## Discussion

While the evaluation on both synthetic and real data showed that locality-sensitive hashing could be used in a promising way to detect signals in mass spectrometry raw data, two challenges are expected when applying the method to real world data.

First, in some mass spectrometry setups so-called chemical noise is present. These are signals that originate in chemical impurities in the measurement workflow and are characterized by repeated occurrences. Thus, the chemical noise is self-similar and would be classified as a “true” signal accordingly by this approach. Since the removal of contaminants can also be performed in subsequent steps in a processing pipeline, this is of lesser concern, but should be kept in mind. Second, signals with a low relative intensity to noise peaks could be lost, as the similarity measure on which the LSH is based drops.

While enabling the evaluation of the proposed approach in a controlled environment, synthetic data cannot fully reflect the behavior of real data. However, as we could not find a gold-standard ground-truth peak-level annotation of data, the evaluation on real data has its own challenges as well. Thus we used the precursor ions selected by the vendor software as a reference to measure the performance of our approach.

Using vendor-selected peaks from a DDA experiment comes with its own challenges. First, the annotated masses correspond only to peptides selected for fragmentation and, second, those peptides likely stem from high-intensity signals. Nonetheless, being able to retrieve those signals with our approach, which is not explicitly based on absolute intensity values, increases confidence in its general applicability.

Additionally, we indicate a potential application for desiotoping and charge state detection. This was demonstrated by our prototypic implementation of a reference-based pattern matching using isotopic patterns following the averagine model. The reference search is not yet perfect, for instance we observed that for some cases the the monoisotopic $\mathrm {m/z}$ value identified is shifted by multiples of $\mathrm {m_n/z}$. Furthermore, charge state identification sometimes fails as well.

Lastly, our implementation is generic enough to be used not only for signal filtering but could also be further extended for additional tasks. Calculating signatures that are locality-sensitive allows for several applications such as: approximate nearest neighbours in constant time, e.g., for database lookup [19], or approximate all near pairs in linear time, e.g., for spectral clustering [21].

## Conclusions

Due to the rapidly growing amount of mass spectrometry data, the analysis of mass spectrometry raw data could greatly benefit from Big Data methods, most notably implying distributed data storage and highly scalable algorithms.

In this study we showed that locality-sensitive hashing is a desirable approach for signal classification in mass spectrometry raw data. It allows for scalability and provides an approach to signal classification that has a strong focus on self-similarity rather than model assumptions as an intrinsic property of the data.

We propose an implementation using a Big Data framework, such as Apache Spark [31], to facilitate testing on many large data sets from different types of mass spectrometry measurements.

## Methods

### Mass axis windows

For the types of mass spectrometry data considered here, the setup of the mass spectrometer gives rise to a hierarchical structure of the data. In the case of LC-IMS-MS, data is continuously acquired as individual scans across the three dimensions. For each retention time several mobility bins are recorded and in turn for each mobility bin the full spectrum along the mass axis is acquired. In order to allow detection of smaller regions of interest, the algorithm works on short compact subsets (intervals) of the mass axis, henceforth referred to as *windows*. One such window, represented by a list of tuples $(\,\mathrm {m}/\mathrm {z},i)$, is the single datum considered by the algorithm for similarity search.

#### Window generation

The set of windows is created by dividing all recorded mass axes into windows. In order to avoid missing an isotopic pattern by distributing it into two windows, a second set of windows that is offset by half a window length is created, such that the mass axes are covered in an overlapping fashion.

The length of the windows should be wide enough to capture a whole isotopic pattern, if the windows are applied in an overlapping fashion and small enough such that the chance of having several isotopic patterns in a window is small. For an assumed pattern length of up to $5 \mathrm {Da}$ a window length of $10 \mathrm {Da}$ is considered useful.

#### Binning

The calculation of a window’s hash value requires the mass axis in a binned form with equally spaced bins. Finding an optimal binning scheme is by no means trivial: a very fine binning resolution makes the algorithm less robust and increases the computational cost, while a very coarse binning resolution yields an increased loss of information. A binning resolution of $0.1\mathrm {m/z}$ was considered suitable as, on the one hand, it still resolves isotopic pattern with up to charge state five (corresponding to a spacing of $0.2 \mathrm {m/z}$) and, on the other hand, keeps the computational load feasible.

### Locality-sensitive hashing

#### Used similarity measure

Two windows $W_{i}$ and $W_{j}$ shall be considered similar when their mass spectra have the same shape but not necessarily the same overall scale. Therefore, the similarity function $s(W_{i},W_{j})$ used is the cosine similarity

1 $$\begin{aligned} s(W_{i},W_{j}) = \frac{\left<\mathbf {I}_{i},\mathbf {I}_{j}\right>}{\Vert \mathbf {I}_{i}\Vert \Vert \mathbf {I}_{j}\Vert }, \end{aligned}$$

where $\mathbf {I}$ denotes the intensity array of a binned window, $\left<\cdot ,\cdot \right>$ the standard scalar product and $\Vert \cdot \Vert$ the Euclidean norm. As a direct consequence from the linearity of the scalar product and norm, the cosine similarity is scale invariant:

2 $$\begin{aligned} s(\alpha W_{i},\beta W_{j}) = \frac{\left<\alpha \mathbf {I}_{i},\beta \mathbf {I}_{j}\right>}{\Vert \alpha \mathbf {I}_{i}\Vert \Vert \beta \mathbf {I}_{j}\Vert } = \frac{\left<\mathbf {I}_{i},\mathbf {I}_{j}\right>}{\Vert \mathbf {I}_{i}\Vert \Vert \mathbf {I}_{j}\Vert } = s(W_{i},W_{j}), \end{aligned}$$

for $\alpha ,\beta >0$. Thus, the findings of the algorithm are independent of the absolute intensity values.

#### Hash function and amplification

The appropriate family of hash functions for the cosine similarity is the random projection hashing [32]. The hash function is given by $h(\cdot ) = {{\,\mathrm{sign}\,}}(\left<\cdot ,r\right>)$ with the components of the vector *r* being random samples from a standard normal distribution.

To control for false positives or false negatives, an augmented LSH with *n* AND connectives and *m* OR connectives is used. This means that instead of computing a single hash function $m\times n$ hash functions are calculated and divided into *m* arrays of length *n*. Two objects $x_{i}$ and $x_{j}$ are now considered collided if all *n* hash functions of a block yield the same value. By choosing *m* and *n* appropriately, a sharp sigmoid shape of the collision probability $P_{m,n}([x_{i},x_{j}])$ can be achieved, which effectively translates into a similarity threshold. Figure 5 shows $P_{m,n}([x_{i},x_{j}])$ for different values of *m* and *n*.

### Intensity thresholding

A very simple and general approach to signal classification is by means of thresholding with respect to a signal-to-noise ratio [33]. If one assumes a global noise estimate, this reduces to a thresholding with respect to the intensity of each peak.

### Data generation

The synthetic data sets consist of windows of length of $10 \mathrm {m/z}$, with a binning resolution of $0.1\mathrm {m/z}$.

In each set, two types of data were created: firstly, windows containing no isotopic patterns and noise only. Secondly, windows containing both isotopic patterns and noise. The label of each peak (“signal”, if it is part of an isotopic pattern, “noise_2”, if it is a noise peak in a window that contains an isotopic pattern, and “noise_1”, if it is a noise peak inside a window without an isotopic pattern being present) was stored for later evaluation. In total 18445 windows per data set were created, of which 14107 contained noise only and 4338 contained both signal and noise.

In the different data sets the intensities of the “true” signals were scaled differently, such that the maximum signal peak has an intensity of 1000, 500, 250, 125, 64, or 32, respectively.

#### Modeling noise

The noise signal is assumed to consist of independently sampled peaks that share the following properties: the number of peaks per window, *k*, is given as

$$\begin{aligned} k \sim \mathrm {Pois}(\lambda _p) + 1, \end{aligned}$$

where $\mathrm {Pois}$ denotes a Poisson distribution with mean $\lambda _p$. Our data was generated using $\lambda _p = 4$.

The location of each peak was sampled uniformly within the window and the intensity value *i* of each peak was sampled from

$$\begin{aligned} i \sim \mathrm {Exp}(\lambda _e), \end{aligned}$$

where $\mathrm {Exp}$ denotes a exponential distribution with mean $\lambda _e^{-1}$. Our data was generated using $\lambda _e = 15$.

#### Modeling isotopic patterns

For the “true” signal the averagine model [11] was used. The monoisotopic peak was placed in the middle of the window and the contribution of the next five peaks was considered. In order to model the repeated occurrences of patterns, two copies with all peaks scaled to half intensities were added. Finally, a noise signal, individually sampled according to description above, was added to every true pattern window.

The monoisotopic masses were ranged from $150 \mathrm {u}$ to $5000 \mathrm {u}$ in steps of $10 \mathrm {u}$. Charge states from 1*e* up to 5*e* were included if the resulting $\mathrm {m/z}$ was in the interval $[150 \mathrm {m/z},2000 \mathrm {m/z}]$.

### Classification by collision

In order to classify windows into “noise” or “signal”, for each window in the data set (several) hash values are computed. Then the number of occurrences of each hash value is counted and all hash values that occurred more then one time are stored in a table, the so-called collision table. A window in question is called “signal” if at least one of its hash values can be found in the collision table, otherwise it is called “noise”.

A single peak in the data set is classified by whether there was a collided window that contains the peak. This is especially important as peaks can be part of several windows due to the overlapping window approach.

### Evaluation on synthetic data

Although the synthetic data provides ground truth labels, a meaningful computation of true-positive and false-negative rates is not straightforward.

Since in our approach all peaks in a window will be assigned the same label, the following problem is caused: when signal and noise are present in a window, peaks with label “noise_2” will be considered “true” as well, which would result in a high false classification rate. For work on real world data however, the inability to remove noise peaks among isotope patterns is typically cured by the fact that subsequent processing steps like feature finding can take the multidimensional signal shape into account, which facilitates the removal of remaining noise peaks.

Thus, in order to learn about the false classification rate of actual interest, peaks with label “noise_2” were not considered for the computation of classification rates both for the intensity thresholding and our approach.

The presence of peaks with label “noise_2” is nevertheless important to test whether whole patterns are missed due to noise in the same window.

### LC-IMS-MS real data set used

A nanoLC-TIMS-MS/MS (DDA-PASEF [8]) analysis of HeLa whole proteome digest was used. HeLa cells were lysed in a urea-based lysis buffer (7 M urea, 2 M thiourea, 5 mM dithiothreitol (DTT), 2$\%$ (w/v) CHAPS) assisted by sonication for 15 min at 4C in high potency using a Bioruptor instrument (Diagenode). Proteins were digested with Trypsin using a filter-aided sample preparation (FASP) [34] as previously detailed [35]. 200 ng of peptide digest were analyzed using a nanoElute UPLC coupled to a TimsTOF PRO MS (Bruker). Peptides injected directly in an Aurora 25 cm x 75 m ID, 1.6 m C18 column (Ionopticks) and separated using a 120 min. gradient method at 400 nL/min. Phase A consisted on water with 0.1$\%$ formic acid and phase B on acetonitrile with 0.1$\%$ formic acid. Sample was injected at 2$\%$ B, lineally increasing to 20$\%$ B at 90 min., 35$\%$ B at 105 min., 95$\%$ at 115 min. and hold at 95$\%$ until 120 min. before re-equilibrating the column at 2$\%$B. The MS was operated in DDA-PASEF mode [8], scanning from 100 to 1700 m/z at the MS dimension and 0.60 to 1.60 1/k0 at the IMS dimension with a 100 ms TIMS ramp. Each 1.17 sec MS cycle comprised one MS1 and 10 MS2 PASEF ramps (frames). The source was operated at 1600 V, with dry gas at 3 L/min and 200C, without nanoBooster gas. The instrument was operated using Compass Hystar version 5.1 and timsControl version 1.1.15 (Bruker). All reagents and solvents used were MS-grade.

### Evaluation on real data

The vendor software returns a list of peptides selected for fragmentation. For these peptides the $\mathrm {m/z}$ coordinate of the monoisotopic peak and apex of ion mobility distribution are given on a frame basis. In the following, we consider the peptides as 2d data points with the corresponding coordinates.

For our approach we built the mass axis windows as described above with a window length of $10\mathrm {m/z}$ from precursor ion frames. Our signal classification returns the mass axis bin and scan of the windows considered signal. We used the center of the window and the scan number as coordinates to represent the found windows as 2d data points as well.

A peptide is considered matched if its nearest neighboring window is closer than 5 units in Manhattan distance. Finally, we calculated the reduction rate, i.e., one minus the fraction of windows considered signal of all windows in a frame. For computing the reduction rate in terms of peaks, we considered all peaks in a signal window to be signal and computed again one minus the fraction of peaks considered signal of all peaks in a frame. Also we computed the fraction of peptides considered matched.

We averaged the results on 50 randomly selected frames, excluding the first 1500 frames from the data set. The whole procedure was repeated for varying *m* and *n*.

### Isotope pattern reference construction and evaluation of reference search

The averagine model as proposed by [11] was used to create all reference patterns. It was sampled with a step size of $10^{-5}$ in a range from $-1$ to $+9$ Da around the monoisotopic peak and afterwards binned to a resolution of $10^{-2}$ Da. This was done for all masses from 150 to 1800 Da and for all charge states from 1 to 5.

We then applied our hashing strategy to those synthetically generated mass spectra the same way it was applied to self-similarity detection. When an unidentified window is now hashed, its key set can be used to select a set of candidate isotope patterns, for which cosine similarity is calculated explicitly. Should a certain threshold of similarity be surpassed (here we used 0.6), the highest scoring reference pattern is returned together with its charge state and monoisotopic mass.

Again we used the list of peptides selected for fragmentation by the vendor software, with the $\mathrm {m/z}$ coordinate of the monoisotopic peak, the apex coordinate of the ion mobility distribution, and now additionally the charge state. In the following, we considered the peptides as 3d data points with the corresponding coordinates. A peptide selected by the vendor software is considered matched if the Manhatten distance to the nearest neighbor in that particular space is less then a threshold of, here, 10 units.

### Scalability study

The scalability study was performed on a single Ubuntu 20.04 LTS machine with AMD Ryzen Threadripper 3990X processor featuring 64 physical cores @2.9 GHz. Overall, enabled hyper-threading allows for the parallel execution of 128 threads. The machine is further equipped with 256 GiB (8x 32 GiB) of DIMM DDR4 @2667 MHz main memory.

As test data 100 randomly chosen MS1 frames of the real data set was used.

## Supplementary Information

**Additional file 1.** Collision probability as a function of m and n for a fixed similarity. Panel A: heatmap view. n is shown on the x-axis, m is shown on the y-axis, the color-coding uses the same scale as the one shown in Panel B. Panel B: surface plot representation. Length trial is synonymous for n and Trials for m.

**Additional file 2.** Receiver operating characteristic curve. This plot shows the relative specificity/sensitivity results of two tested signal detection methods LSH and noise threshold (SNR). The dashed line is for reference and shows the results of random guessing. The different panels show different maximum signal intensities I: For Panel A I = 500, for Panel B I = 250, for Panel C I = 125, for Panel D I = 64, and for Panel E I = 500.

## Data Availability

Data and code is available at https://github.com/hildebrandtlab/mzBucket

## Footnotes

1 Note that the number of windows in the data set influences the collision probabilities as well.
