# Supplementary material for: Locality-sensitive hashing enables efficient and scalable signal classification in high-throughput mass spectrometry raw data
Source: BMC Bioinformatics. 2022 Jul 20;23:287. doi: 10.1186/s12859-022-04833-5 (PMC9301846; doi:10.1186/s12859-022-04833-5)
Supplement: Supplementary file 1 — Additional file 1. Collision probability as a function of m and n for a fixed similarity. Panel A: heatmap view. n is shown on the x-axis, m is shown on the y-axis, the color-coding uses the same scale as the one shown in Panel B. Panel B: surface plot representation. Length trial is synonymous for n and Trials for m. [file 12859_2022_4833_MOESM1_ESM.pdf]

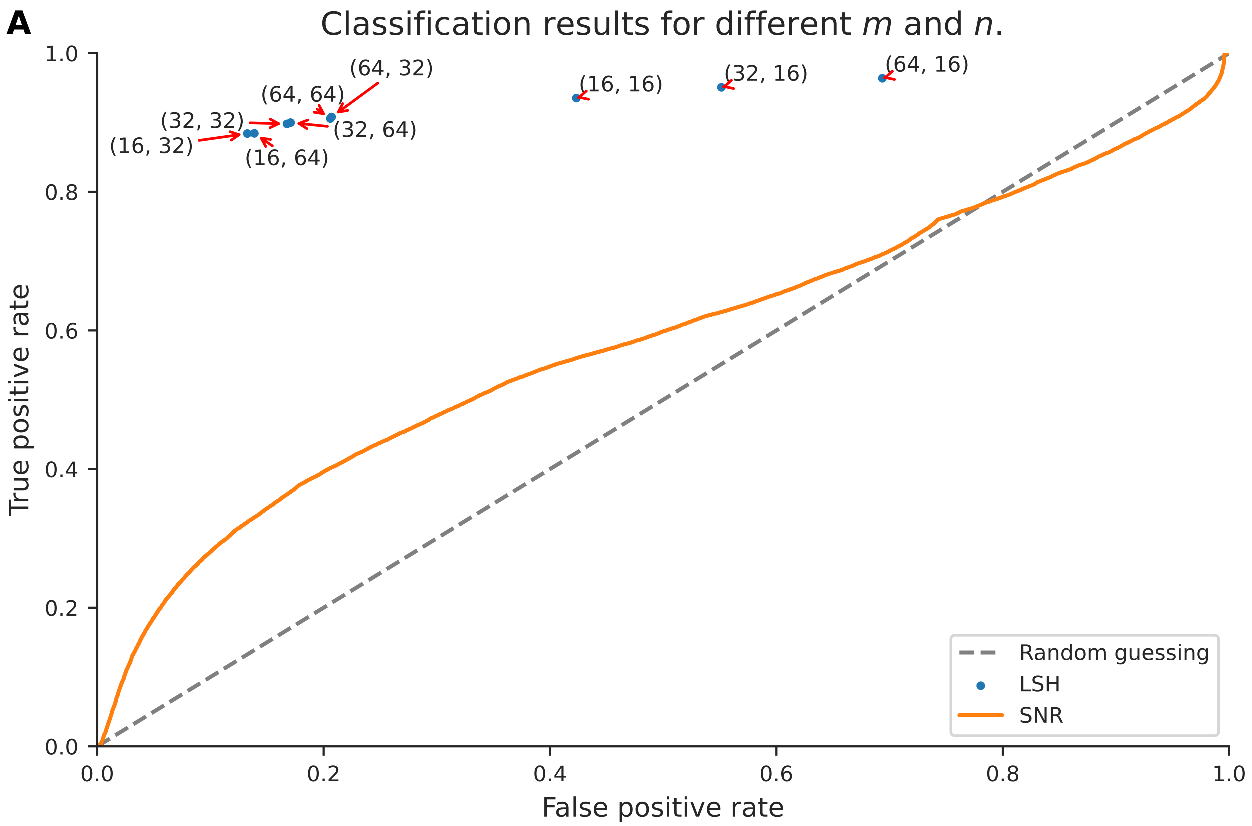

A
Classification results for different *m* and *n*.
(64, 32)
(64, 64)
(32, 32)
(16, 32)
(32, 64)
(16, 64)
(16, 16)
(32, 16)
(64, 16)
1.0
0.8
0.6
0.4
0.2
0.0
True positive rate
0.0
0.2
0.4
0.6
0.8
1.0
False positive rate
Random guessing
LSH
SNR

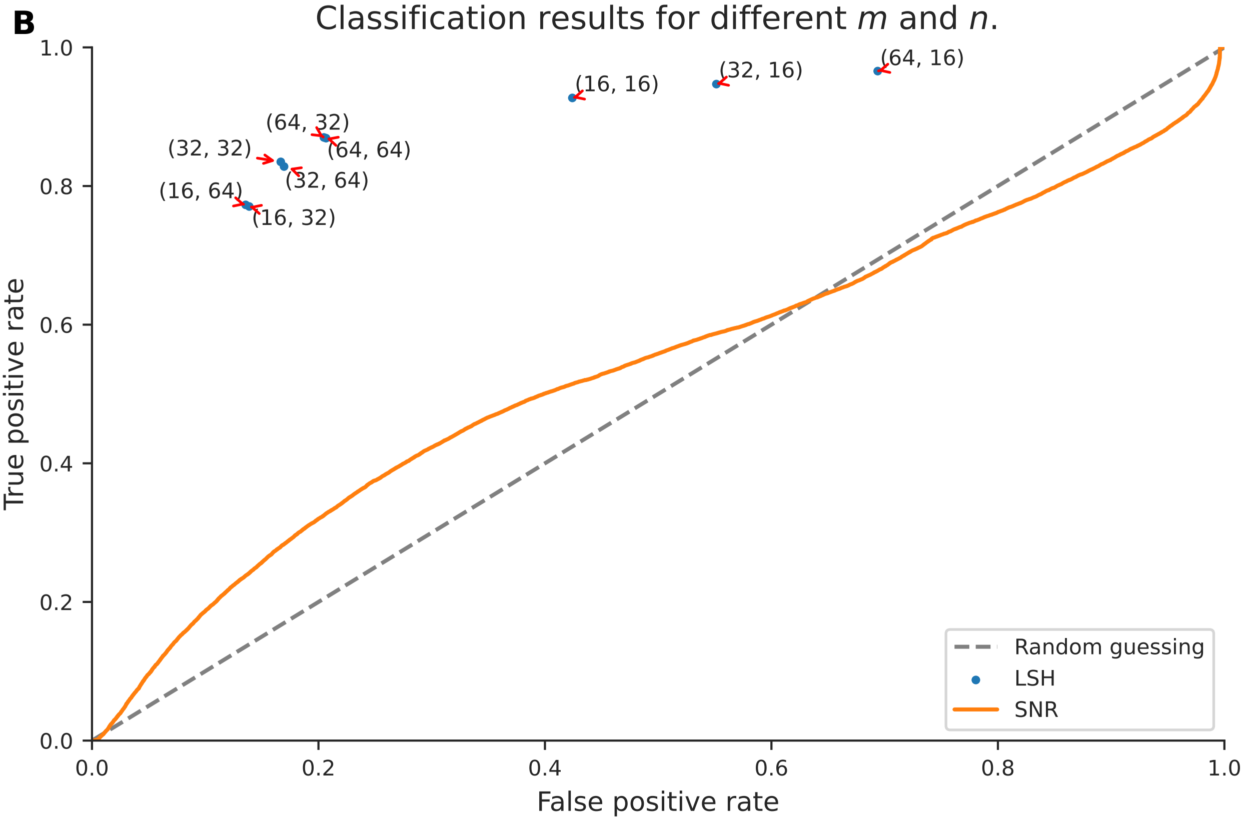
B
Classification results for different m and n.
(64, 16)
(32, 16)
(16, 16)
(64, 32)
(32, 32)
(64, 64)
(32, 64)
(16, 64)
(16, 32)
1.0
0.8
0.6
0.4
0.2
0.0
True positive rate
0.0
0.2
0.4
0.6
0.8
1.0
False positive rate
Random guessing
LSH
SNR

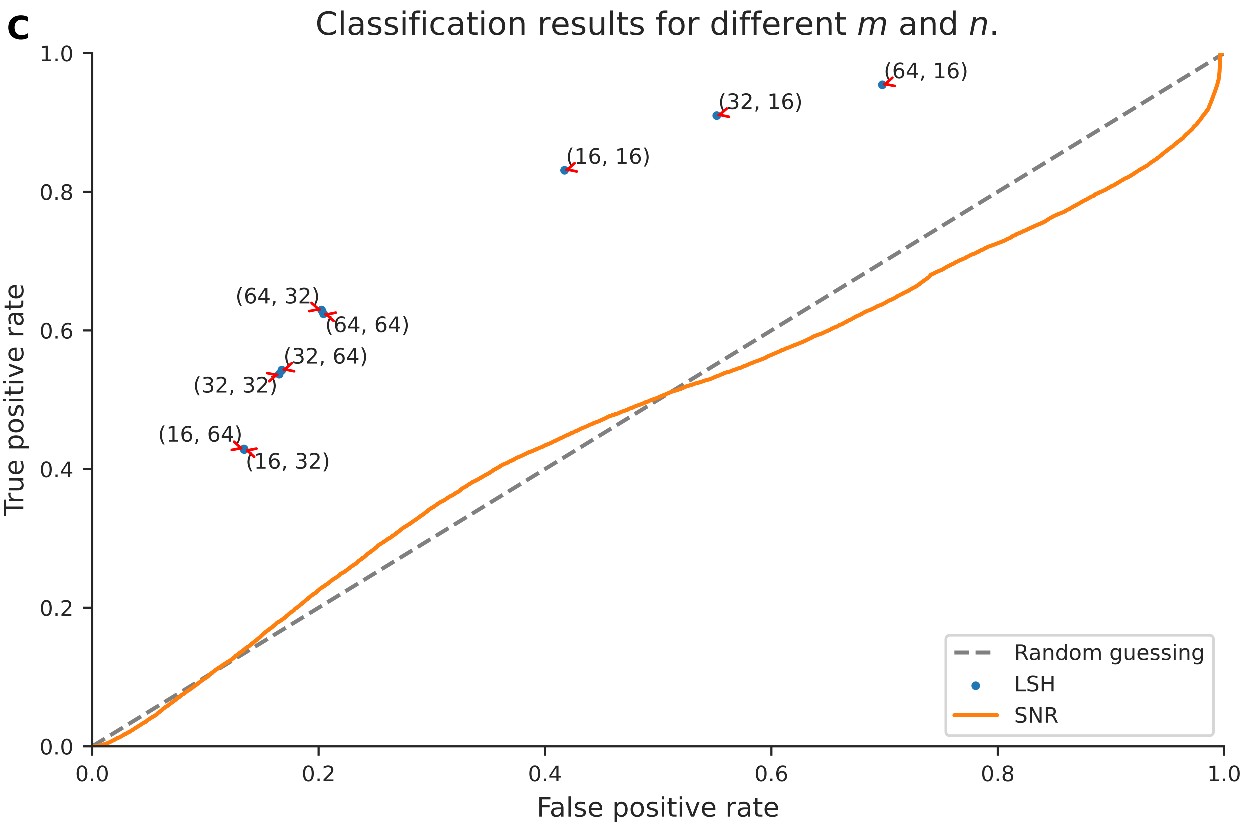
c
Classification results for different m and n.
(64, 16)
(32, 16)
(16, 16)
(64, 32)
(64, 64)
(32, 64)
(32, 32)
(16, 64)
(16, 32)
Random guessing
LSH
SNR
True positive rate
False positive rate
0.0
0.2
0.4
0.6
0.8
1.0

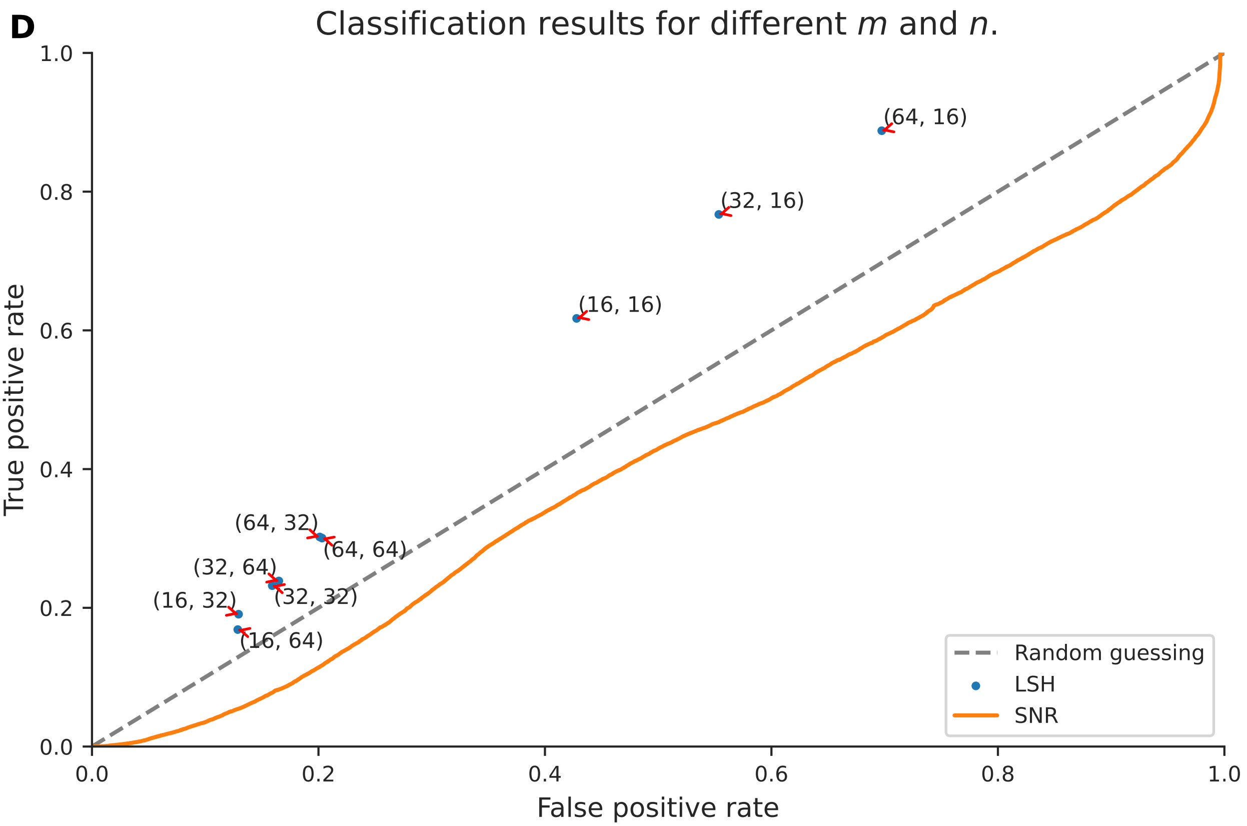
D
Classification results for different m and n.
1.0
0.8
0.6
0.4
0.2
0.0
True positive rate
0.0
0.2
0.4
0.6
0.8
1.0
False positive rate
(64, 16)
(32, 16)
(16, 16)
(64, 32)
(64, 64)
(32, 64)
(32, 32)
(16, 32)
(16, 64)
Random guessing
LSH
SNR

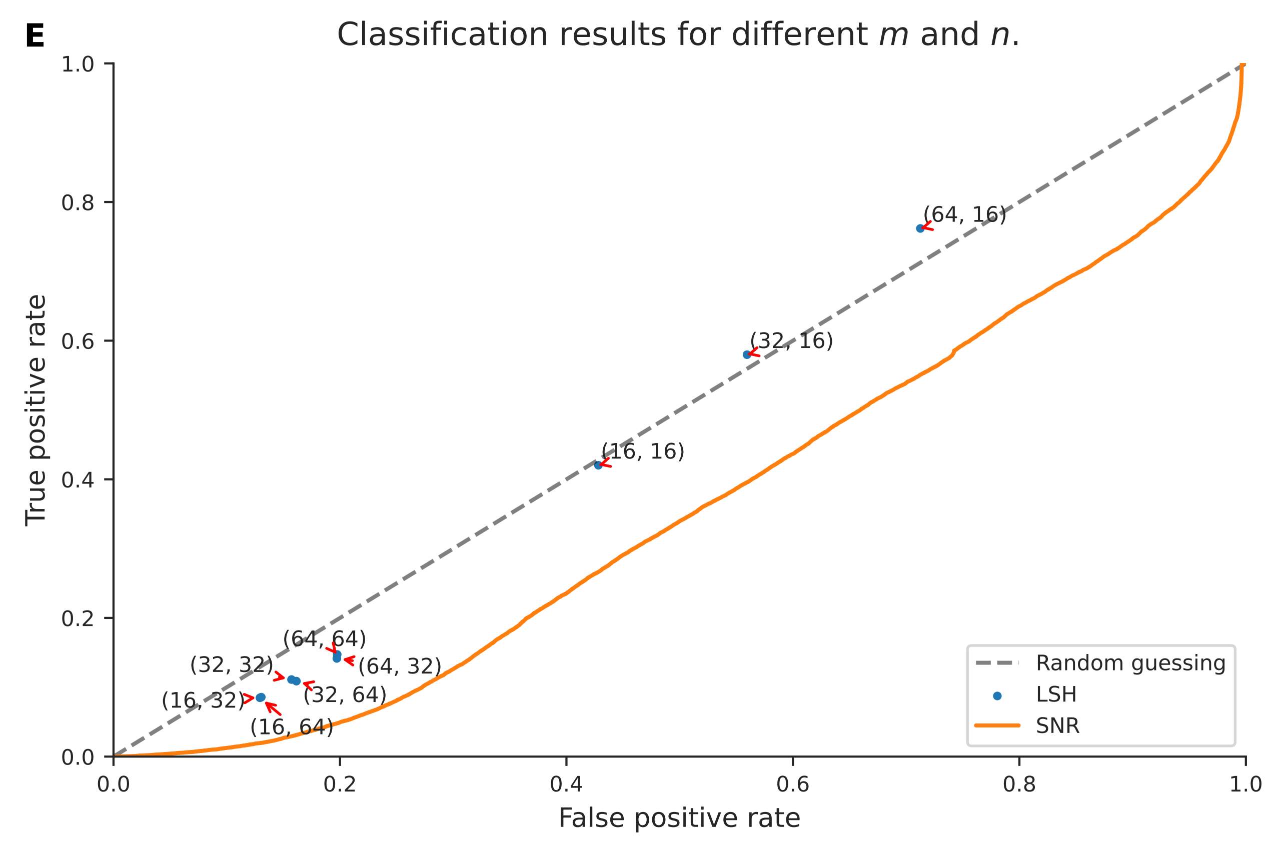
E
Classification results for different m and n.
True positive rate
False positive rate
0.0
0.2
0.4
0.6
0.8
1.0
(64, 16)
(32, 16)
(16, 16)
(64, 64)
(64, 32)
(32, 32)
(32, 64)
(16, 32)
(16, 64)
Random guessing
LSH
SNR
