# Supplementary figures and images for: Locality-sensitive hashing enables efficient and scalable signal classification in high-throughput mass spectrometry raw data
Source: BMC Bioinformatics. 2022 Jul 20;23:287. doi: 10.1186/s12859-022-04833-5 (PMC9301846; doi:10.1186/s12859-022-04833-5)

**A**

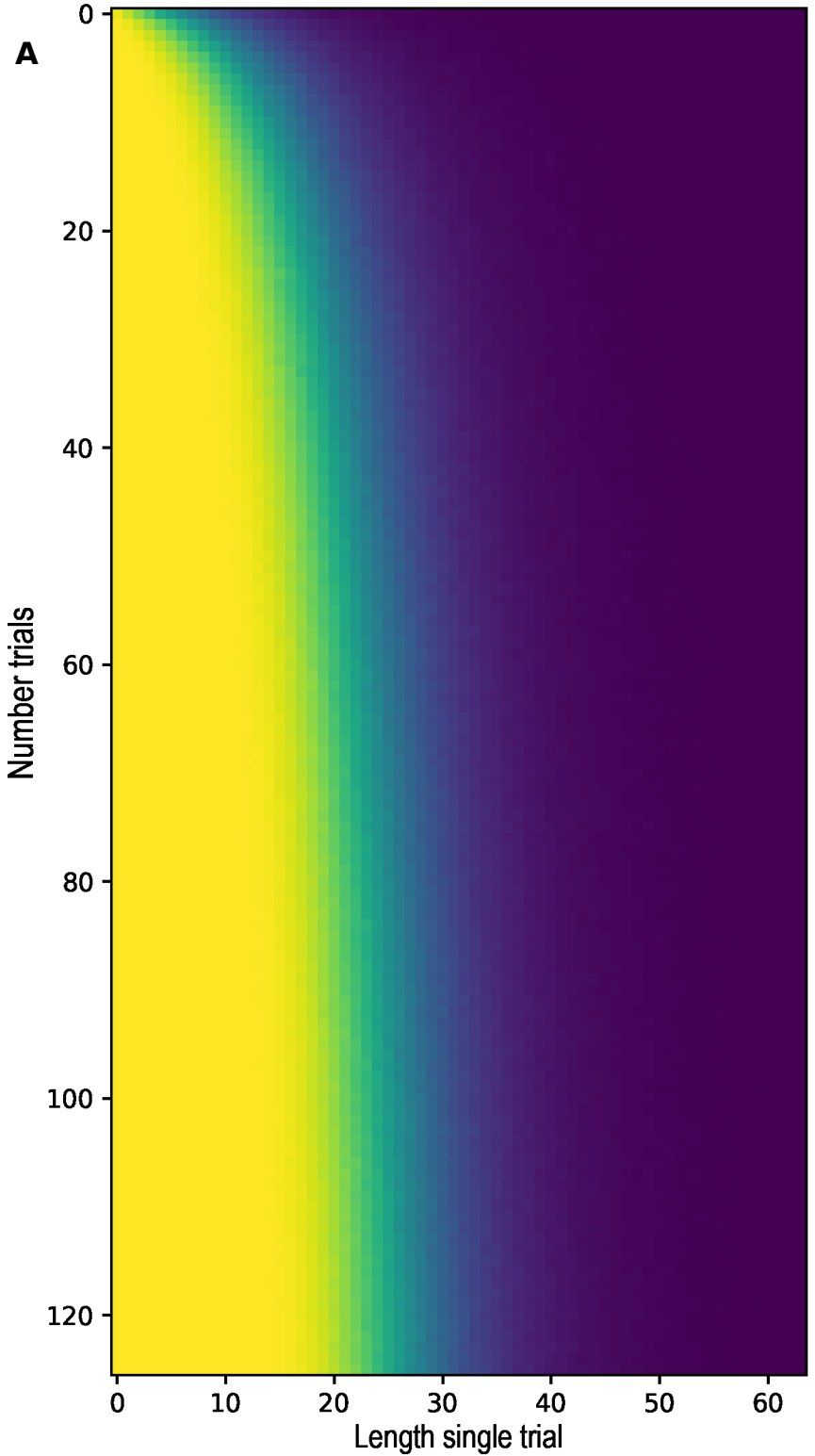

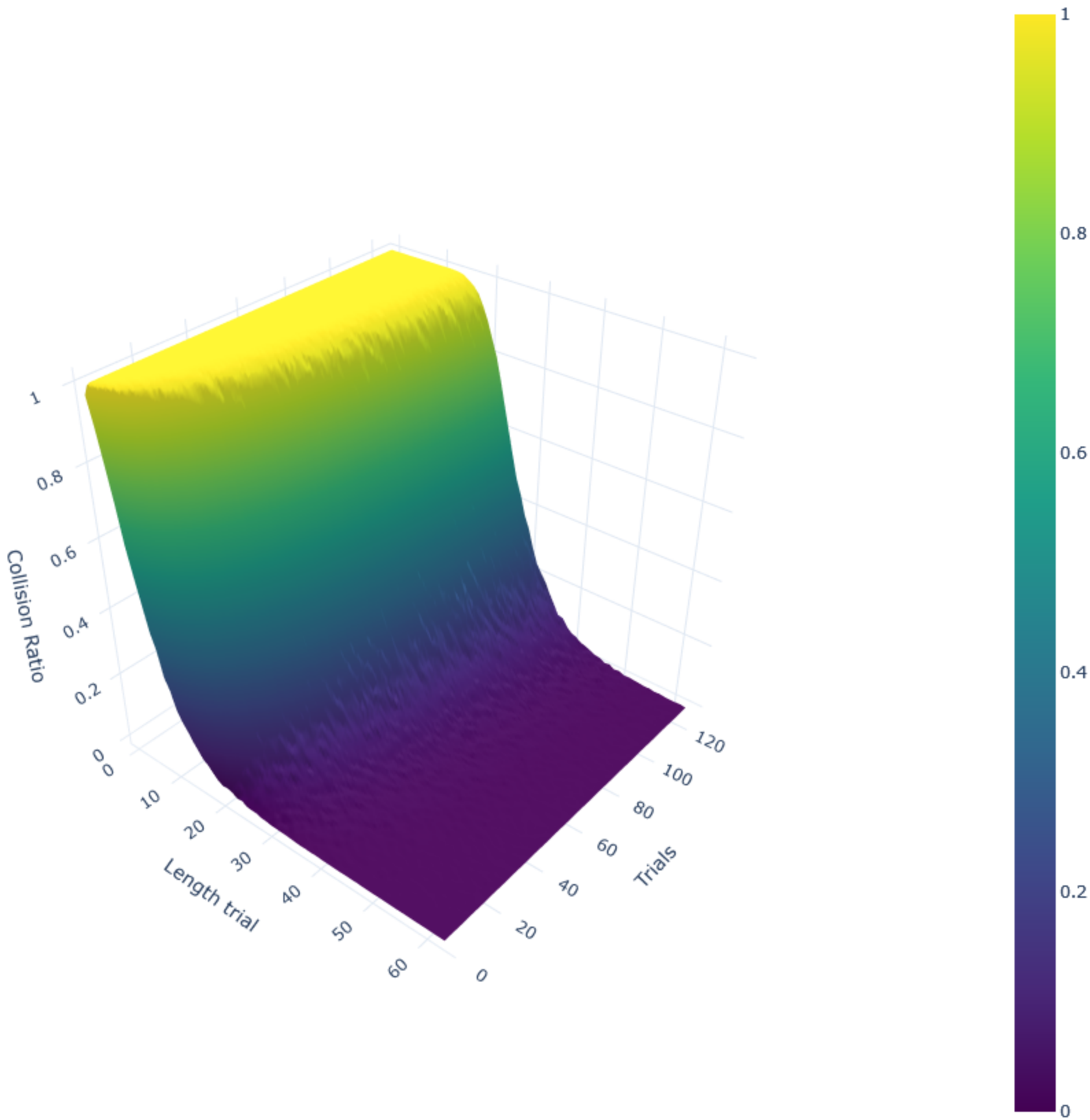

Supplement: Supplementary file 2 — Additional file 2. Receiver operating characteristic curve. This plot shows the relative specificity/sensitivity results of two tested signal detection methods LSH and noise threshold (SNR). The dashed line is for reference and shows the results of random guessing. The different panels show different maximum signal intensities I: For Panel A I = 500, for Panel B I = 250, for Panel C I = 125, for Panel D I = 64, and for Panel E I = 500. [file 12859_2022_4833_MOESM2_ESM.pdf]
